# Possible Role of Cytochrome P450 1B1 in the Mechanism of Gemcitabine Resistance in Pancreatic Cancer

**DOI:** 10.3390/biomedicines9101396

**Published:** 2021-10-05

**Authors:** Erica Yada, Rika Kasajima, Atsushi Niida, Seiya Imoto, Satoru Miyano, Yohei Miyagi, Tetsuro Sasada, Satoshi Wada

**Affiliations:** 1 Department of Cancer Immunotherapy, Kanagawa Cancer Center Research Institute, Yokohama 241-8515, Japan; erika.ya@gancen.asahi.yokohama.jp (E.Y.); tsasada@kcch.jp (T.S.); 2 Molecular Pathology and Genetics Division, Kanagawa Cancer Center Research Institute, Yokohama 241-8515, Japan; rkasajima@gancen.asahi.yokohama.jp (R.K.); miyagi@gancen.asahi.yokohama.jp (Y.M.); 3 Division of Health Medical Data Science, Health Intelligence Center, Institute of Medical Science, The University of Tokyo, Minato-ku, Tokyo 108-8639, Japan; imoto@ims.u-tokyo.ac.jp; 4 Division of Health Medical Computational Science, Health Intelligence Center, Institute of Medical Science, The University of Tokyo, Minato-ku, Tokyo 108-8639, Japan; aniida@ims.u-tokyo.ac.jp (A.N.); miyano@ims.u-tokyo.ac.jp (S.M.); 5 Department of Clinical Diagnostic Oncology, Clinical Research Institute for Clinical Pharmacology & Therapeutics, Showa University, 6-11-11 Kitakarasuyama, Setagaya-ku, Tokyo 157-8577, Japan; 6 Department of Medicine, Division of Medical Oncology, School of Medicine, Showa University, Shinagawa-ku, Tokyo 142-8666, Japan

**Keywords:** gemcitabine, PDX model, pancreatic cancer, patient-derived xenograft, RNA sequencing

## Abstract

Patient-derived xenograft models reportedly represent original tumor morphology and gene mutation profiles. In addition, patient-derived xenografts are expected to recapitulate the parental tumor drug responses. In this study, we analyzed the pathways involved in gemcitabine resistance using patient-derived xenograft models of pancreatic cancer. The patient-derived xenograft models were established using samples from patients with pancreatic cancer. The models were treated with gemcitabine to better understand the mechanism of resistance to this anti-cancer drug. We performed comparative gene analysis through the next-generation sequencing of tumor tissues from gemcitabine-treated or non-treated patient-derived xenograft mice and gene set enrichment analysis to analyze mRNA profiling data. Pathway analysis of gemcitabine-treated patient-derived xenografts disclosed the upregulation of multiple gene sets and identified several specific gene pathways that could potentially be related to gemcitabine resistance in pancreatic cancer. Further, we conducted an in vitro analysis to validate these results. The mRNA expression of cytochrome P450 1B1 and cytochrome P450 2A6 was upregulated in a concentration-dependent manner following gemcitabine treatment. Moreover, the sensitivity to gemcitabine increased, and viable cells were decreased by the cytochrome P450 1B1 inhibitor, indicating that the cytochrome P450 1B1 pathway may be related to gemcitabine resistance in pancreatic cancer.

## 1. Introduction

A myriad of cancer cell lines has already been used in biomedical research. However, these cell lines are cultured under artificial conditions and do not necessarily reflect physiological cancer cell kinetics. Therefore, xenografts such as cell line-derived xenografts (CDX) or cancer cell line xenografts (CCLX), which can be transplanted into immunodeficient mice, are widely used in cancer research [1]. CDX provides the advantage of studying disease progression in the physiological environment of mice. However, this system has the disadvantage that the cell lines used to establish CDX have already adapted to in vitro growth conditions. Furthermore, CDX models cannot reconstruct the stroma in the cancer microenvironment. Patient-derived xenograft (PDX) mouse models have recently attracted growing attention, as they represent a potential solution to these problems [2]. To this end, we established 10 PDX model lines for pancreatic cancer.

Pancreatic cancer is one of the most fatal malignancies. It is the fourth leading cause of cancer-related deaths among both men and women [3]. The 5-year survival rate of pancreatic cancer is only approximately 7–8% in the United States, which seems to be due to late-stage diagnosis [4]. Consequently, many patients are ineligible for surgery and have limited chemotherapeutic options. Therefore, the development of new therapeutic approaches for pancreatic cancer remains a priority in the basic, pre-clinical, and clinical cancer research fields. Gemcitabine (GEM) has been widely used as an anticancer chemotherapeutic agent for various solid tumors. After GEM is transported into cells, it is phosphorylated into GEM monophosphate (dFdCMP) by the deoxycytidine kinase (dCK) and is subsequently phosphorylated to GEM diphosphate (dFdCDP) by the pyrimidine nucleoside monophosphate kinase (NMPK) and to gemcitabine triphosphate (dFdCTP) by the nucleoside diphosphate kinase (NDPK). The major cellular metabolite of gemcitabine, dFdCTP, acts as a competitive substrate for deoxycytidine triphosphate (dCTP). This allows dFdCTP to be incorporated into DNA during replication, thus inhibiting the chain elongation of DNA and causing cell death by apoptosis [5]. GEM has been the standard treatment for advanced stages of pancreatic cancer for a long time; however, cancers quickly develop resistance to this drug [6]. Hence, the resistance to this anti-cancer drug presents a major problem in the field of pancreatic cancer research, and understanding the mechanism of such resistance is an urgent issue.

Several studies have reported different mechanisms of GEM resistance. For instance, tumor-associated macrophages and myofibroblasts express insulin-like growth factors (IGFs) in the pancreatic tumor microenvironment, and stromal-derived IGFs enhance the resistance of pancreatic cancer cells to chemotherapy [7]. Shukla et al. reported that the inhibition of glycolysis or pyrimidine biosynthesis leads to increased GEM sensitivity [8]. Furthermore, it has been demonstrated that various transcription factors, enzymes, and signaling pathways involved in nucleoside metabolism are involved in the development of chemoresistance to GEM [7,8,9,10]. However, the mechanism behind the development of GEM resistance remains unclear.

Many previous studies on GEM resistance have used cell lines as their model. In this study, we analyzed the genetic pathway of the GEM resistance mechanism and aimed to identify the molecules related to GEM resistance using the seven established PDX models for pancreatic cancer.

## 2. Materials and Methods

### 2.1. Animals

NSG mice (6–12 weeks old) obtained from the Jackson laboratory (Sacramento, CA, USA) and Charles River Laboratories Japan (Yokohama, Kanagawa, Japan) were used in this study. All of the animals were housed in plastic cages in a pathogen-free environment at a temperature of 22 ± 1 °C with 45 ± 10% humidity and a 12 h light/12 h dark cycle. All of the experiments involving laboratory animals were performed in accordance with the care and use guidelines of the Kanagawa Cancer Center Research Institute.

### 2.2. Tumor Tissues for Transplantation

The surgically removed fresh tumor tissues for transplantation were obtained from the Kanagawa Cancer Center, with the patients’ providing written informed consent for the study. The study was approved by the Research Ethics Committee of the Kanagawa Cancer Center (approval no. E-176 and E-244).

### 2.3. Establishment of Xenografts

We transplanted several pieces of the surgically removed tissues as described previously [11,12]. None of the patients from whom the samples were obtained received chemotherapy. Briefly, after the surgery, the fresh tumor tissues were divided into very small pieces using scissors or were minced under sterile conditions. It was important to use an additive-free medium or phosphate-buffered saline (PBS) to avoid drying out the tissue. A small incision was made at the back (lower part) of the mouse, close to the hip region, and a transplant needle was inserted until the tip reached the dorsal subcutaneous area of the upper part of the back, and the skin was closed. This transplantation distance was necessary to prevent engraftment outflow. After the expansion of the engrafted mass, the xenograft tumor was re-transplanted for further expansion in another immunodeficient mouse following the same procedure. The possibility of tumor tissue storage is one of the main advantages of PDX establishment, as tumor tissues are submerged in a cryopreservation medium, such as Cellbanker 1 (Zenoaq, Fukushima, Japan), and are then stored in liquid nitrogen. Frozen tumor tissues can be thawed and used for re-transplantation and expansion. PDX lines were established after three tumor tissue passages. The established PDX lines were used in subsequent experiments.

### 2.4. Gemcitabine (GEM) Administration

GEM was purchased from Eli Lilly (Kobe, Japan). GEM was administered to mice via intra-abdominal injections twice a week. One administration was skipped after three injection sessions. The administration was maintained until the mice were sacrificed. The dose applied to the NSG mice was determined in preliminary experiments.

### 2.5. Immunohistochemistry

The sample tissues were fixed in 10% formalin and were embedded in paraffin (FFPE). Four micrometer thick sections were prepared and were subjected to standard hematoxylin and eosin (H&E) staining or immunohistochemistry. After deparaffinization and washing in PBS, endogenous peroxidase activity was inactivated by treatment with 0.3% H_2_O_2_ for 30 min. Primary anti-HLA class I-A, B, and C antibodies (Hokudo, Sapporo, Japan) were applied at a 1:500 dilution and were incubated for 60 min. Simple Stain MAX PO (M) (Nichirei Biosciences) was used for secondary staining and incubated for 1 h. After washing, the cells were visualized with DAB and H_2_O_2_.

### 2.6. RNA Extraction and High-Throughput Sequencing

The tumor tissues were cut into small pieces and were subsequently frozen in liquid nitrogen within the shortest delay possible after the tumor-bearing mice were sacrificed. The frozen tissue was homogenized using a cryo-press (Microtec, Funabashi, Chiba, Japan). Total RNA was extracted from homogenized tissue and was purified using a ZR-Duet DNA/RNA MiniPrep kit (Zymo Research, Irvine, CA, USA). RNA quality was assessed using an Agilent 2100 Bioanalyzer (Agilent Technologies, Santa Clara, CA, USA). cDNA libraries were established from the RNA samples, and the libraries were sequenced using an Illumina HiSeq2000 platform at BGI (Shenzhen, China). Approximately 5 GB of raw reads were generated for each sample.

RNA Seq data from PDXs were processed with Xenome (version 1.0.1) (https://github.com/data61/gossamer/blob/master/docs/xenome.md; accessed on 10 May 2021) [13] to classify the raw sequenced reads into human (hg19) or mouse (mm9) reads. Transcript expression was quantified using Salmon (version 0.6.0) (https://github.com/COMBINE-lab/salmon; accessed on 22 July 2021) [14]. Furthermore, we generated a heatmap and cluster of differentially expressed genes in RNAseq using R (version 3.2.0) (https://www.r-project.org; accessed on 22 July 2021).

### 2.7. Gene Set Enrichment Analysis (GSEA)

GSEA was performed using GSEA software version 2.0 provided by the BROAD institute (http://www.gsea-msigdb.org/gsea/index.jsp; accessed on 10 May 2021) using predefined gene sets from the Molecular Signatures Database (MSigDB v5.0) [15]. The gene ontology (GO) term gene set v5.1 (including 5917 gene sets) was chosen for GO enrichment analysis. The enrichment score (ES) normalized the ES (NES), *p* value, and false discovery rate (FDR). Q values were obtained from the GSEA output reports, which were then used to rank the gene sets.

### 2.8. Cell Culture

MIA PaCa-2 and KP4 cells were purchased from the American Type Culture Collection (Manassas, VA, USA). These cells were cultured in RPMI 1640 (Invitrogen, Carlsbad, CA, USA) and DMEM (Invitrogen, Carlsbad, CA, USA), respectively, at 37 °C in a humidified 5% CO2 atmosphere. Both media were supplemented with 10% fetal bovine serum (FBS; GE Healthcare, Uppsala, Sweden), 50 U/mL penicillin, and 50 µg/mL streptomycin sulfate. Cells were seeded in 6-well plates for 24 h before transfection. After 24 h of growth, GEM, GEM, and alizarin (Nacalai Tesque, Kyoto, Japan) were added at various concentrations. RNA was extracted using the following method 48 h after GEM stimulation.

### 2.9. Quantitative Real-Time PCR (RT-qPCR)

RNA was extracted using an RNAeasy Mini Kit (Qiagen, Hilden, Germany). RNA (1 µg) was reverse transcribed into cDNA using a SuperScript III kit (Invitrogen), according to the manufacturer’s protocols. The following primer sets were used for RT-qPCR: forward primer for human *GAPDH* cDNA 5′-GCA AAT TCC ATG GCA CCG T-3′, reverse primer for human *GAPDH* cDNA 5′-TCG CCC CAC TTG ATT TTG G-3′; forward primer for human *CYP1B1* cDNA 5′-CAC TGC CAA CAC CTC TGT CT-3′, reverse primer for human *CYP1B1* cDNA 5′-GGT CCT TGT TGA TGA GGC CA-3′; and forward primer for human *CYP2A6* cDNA 5′-CAA GAC CGG GCT TGG GAG-3′, reverse primer for human *CYP2A6* cDNA 5′-ATC AAG GTG AAC TGA GCC GC-3′. RT-qPCR was performed on a Light Cycler 480 II device (Roche, Basel, Switzerland) using SYBR Green Mastermix (Roche). For the detection of cDNA, one activation step at 95 °C for 10 min was followed by 50 cycles of denaturation at 95 °C for 15 s and annealing/extension at 60 °C for 30 s.

### 2.10. Statistical Analysis

All statistically significant data were analyzed using JMP Pro 14.0 software (SAS Institute Inc., Cary, NC, USA). qPCR gene expression data were evaluated using Student’s *t*-test. The results with a *p*-value of less than 0.05 were considered to be statistically significant.

## 3. Results

### 3.1. Establishment of Pancreatic Cancer PDXs and the Anti-Tumor Effects of GEM Treatment

**Figure 1:**
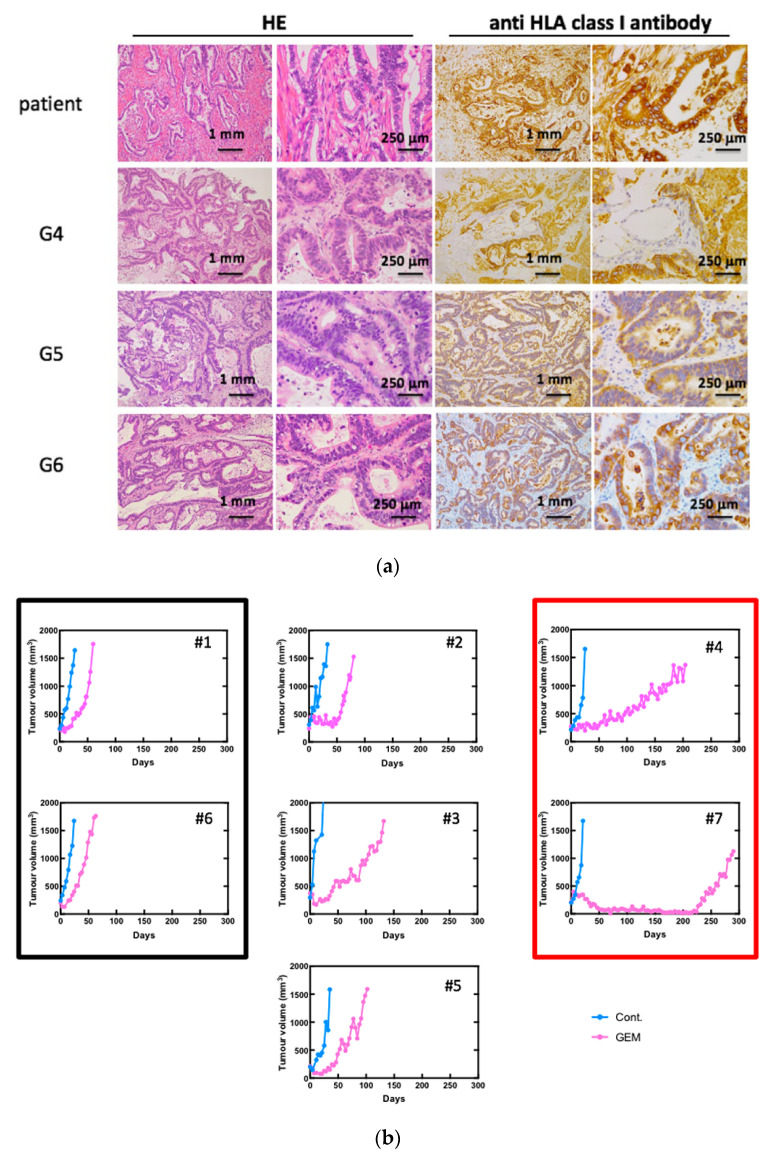
Gemcitabine (GEM)-treated pancreatic cancer patient-derived xenograft (PDX) mice. (**a**) Establishment of PDX mice. Morphological characteristics were well preserved in xenograft tumors in NSG mice even at G6. Hematoxylin and eosin (H&E) staining and immunohistochemistry for anti-HLA class I antibody are shown. (**b**) Tumor growth curve after treatment of GEM. Seven PDX lines were treated with GEM after tumor volume exceeded 200 mm^3^. Different PDXs had different sensitivities for GEM. PDX mice that had high sensitivity for GEM are in the red box, and those with low sensitivity are in the black box. #1–#7 is shown as the name of the xenograft.

We established 10 PDX lines, as previously reported [16]. The morphological characteristics were examined using standard hematoxylin and eosin (H&E) staining and immunohistochemical staining for HLA class I (Figure 1a). We referred to the mice engrafted with a patient-derived tissue as a “G1 mouse” and mice engrafted with G1 mouse-derived tumor pieces as a “G2 mouse” Our results showed that morphology had even been preserved in G6 mice. The human origin of the investigated tissue samples was confirmed using specific anti-HLA class I antibodies. Seven PDX mouse lines were treated with GEM after tumor engraftment to identify chemoresistant genes according to a previously described protocol [16]. Different PDXs had different sensitivities to GEM (Figure 1b). Next, we performed RNA sequencing on both the control and GEM-treated samples that had been efficiently affected by the treatment.

### 3.2. RNA Sequencing and Gene Pathway Analysis

**Figure 2:**
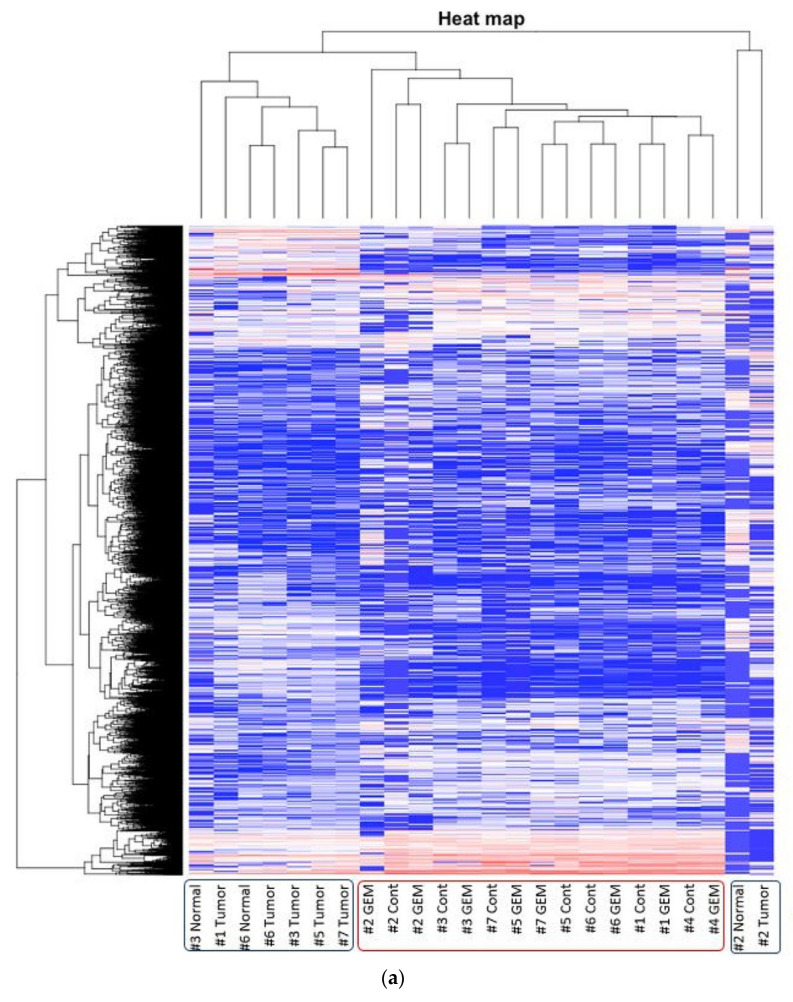

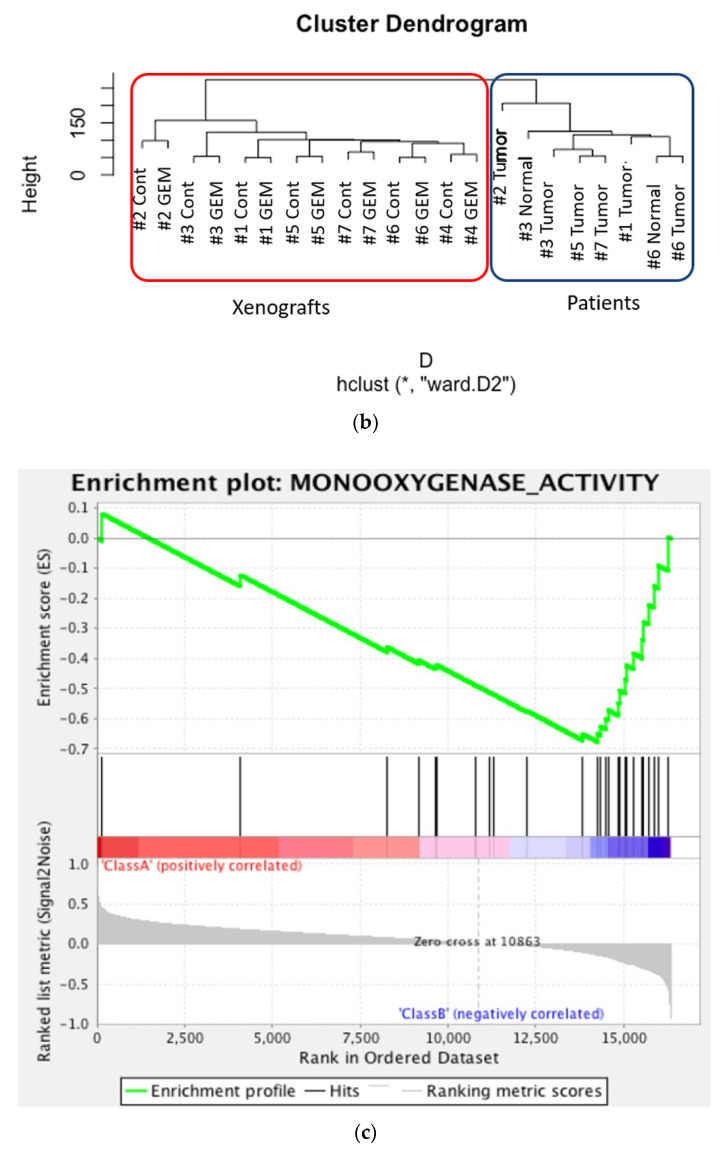

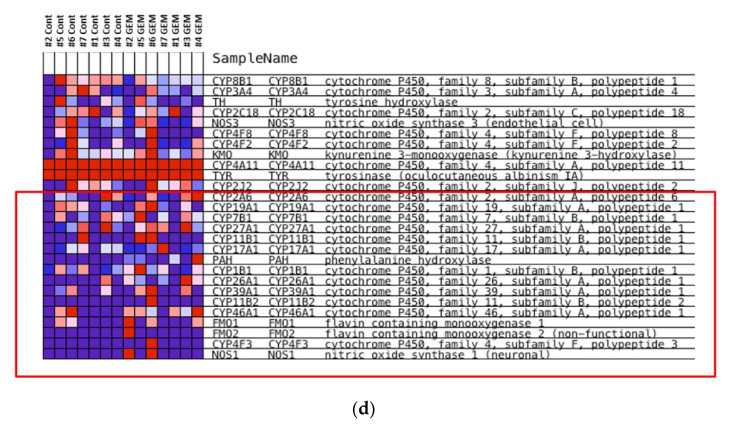
RNA-seq was performed using gemcitabine (GEM)-treated tumors. (**a**) Heat map of RNA expression. Expression values are represented as colors, where the range of colors (red, pink, light blue, and dark blue) shows the range of expression values (high, moderate, low, and lowest), respectively. Patient-derived xenograft (PDX) samples are in the red box, and samples obtained directly from patients are in the blue box. Cont is control sample. (**b**) Cluster dendrogram of (**a**). The height axis displays the distance between observations and/or clusters. The horizontal bars indicate the point at which two clusters/observations merged. PDX samples are in the red box, and samples obtained directly from patients are in the blue box. (**c**) GSEA plot depicting the enrichment of genes upregulated in monooxygenase activity. We analyzed whether the genes whose expression differed between the control and GEM were biased to a specific gene set. (**d**) Heatmap and the list of the genes involved in monooxygenase activity. Among the genes involved in monooxygenase activity, genes within the red lines were upregulated in the GEM-treated group. #1–#7 is shown as the name of the xenograft.

The heat map was generated from the RNA sequencing data and expressed the gene expression values (Figure 2a). Similar RNA expression patterns were observed between GEM-treated and control samples derived from the same patient’s tumor. There were higher individual differences than differences due to treatment (Figure 2b). To detect whether genes were differentially expressed between control samples and GEM-treated samples, Student’s *t*-test and the Wilcoxon signed rank test were performed. However, there were no significant differences between the two groups. Next, we investigated the potentially altered pathways in the samples. To this end, we performed GSEA between the control and GEM-treated groups. As shown in Table 1, the gene groups with an FDR q value less than 0.1 were found to be significantly upregulated. We found the enrichment of the genes involved in monooxygenase activity in the GEM-treated group (Table 1, Figure 2c). A list of the genes involved in monooxygenase activity is shown in Figure 2d. Among the genes involved in monooxygenase activity, the genes within the red lines were upregulated in the GEM-treated group.

### 3.3. Identification of the Key Molecule of GEM Resistance in Pancreatic Cancer

**Figure 3:**
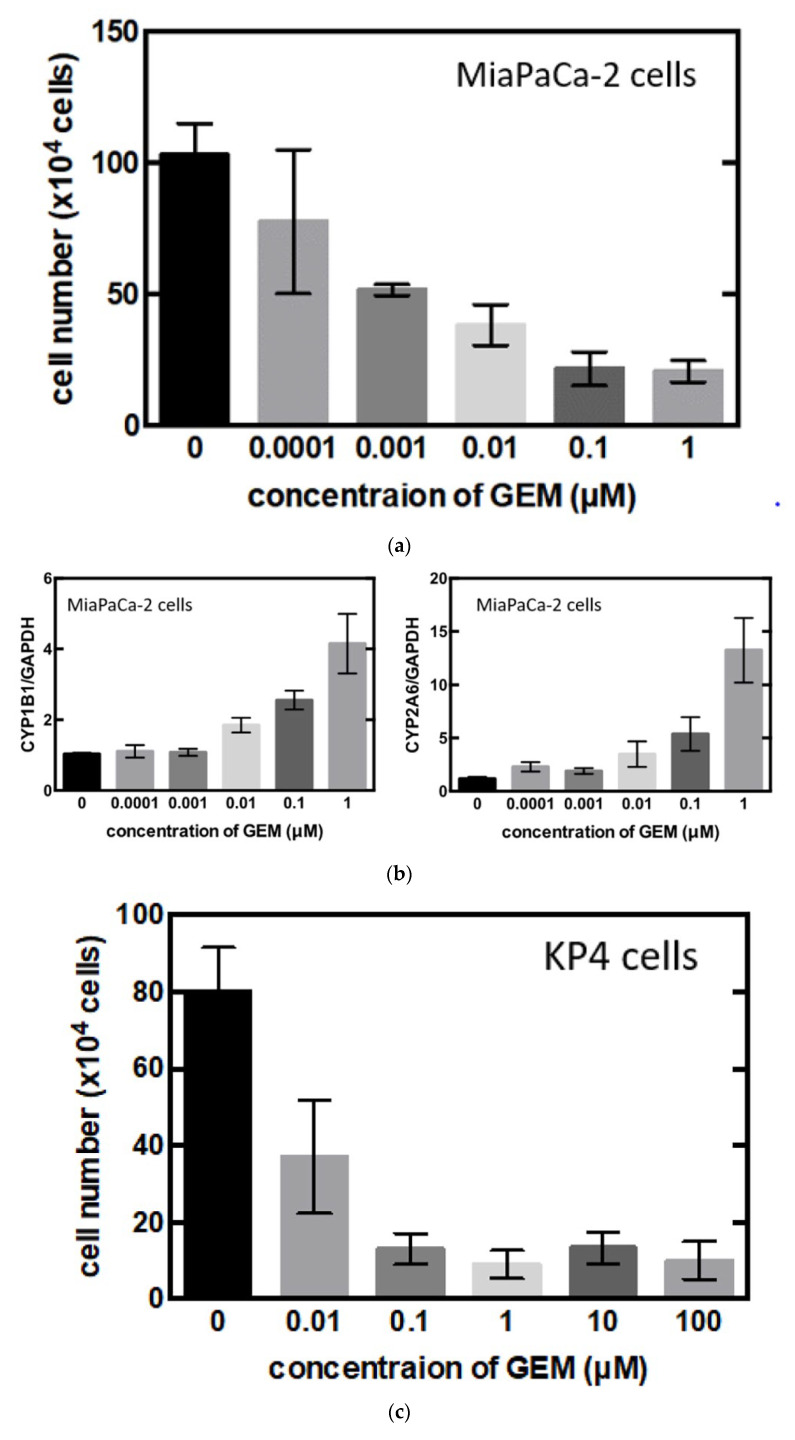

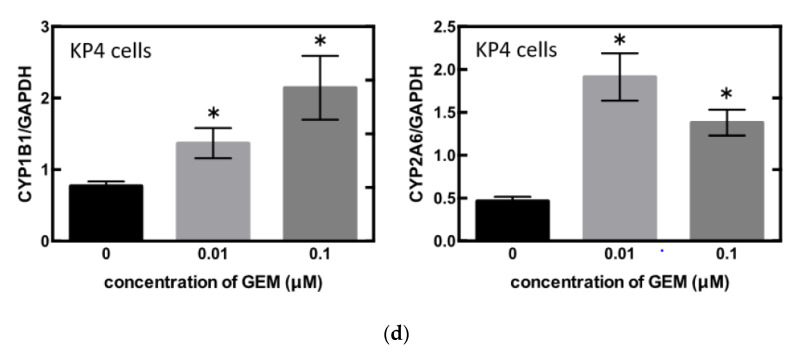
(**a**) MiaPaCa-2 cells were treated with gemcitabine (GEM) 0–1 µM for 48 h, and the cell number was counted. (**b**) MiaPaCa-2 cells were treated with GEM, and after 48 h, RNA was extracted from the cells, and qPCR was performed using CYP1B1 or CYP2A6 primers. (**c**) KP4 cells were treated with GEM 0–1 µM for 48 h, and the cell number was counted. (**d**) KP4 cells were treated GEM, and after 48 h, RNA was extracted from the cells, and qPCR was performed using CYP1B1 or CYP2A6 primers. All data are represented as mean ± SEM, *n* = 3. * *p* < 0.05.

To identify the central compound responsible for GEM resistance in pancreatic cancer, we examined the proliferation of pancreatic cancer cell lines in vitro. The cell number decreased in a concentration-dependent manner following GEM treatment in the MiaPaCa-2 cells (Figure 3a). Conversely, the mRNA expression of CYP1B1 and CYP2A6 increased upon GEM treatment in a concentration-dependent manner (Figure 3b). However, no other expression changes were observed among the genes shown in Figure 2d. We also conducted similar experiments in another pancreatic cancer cell line, KP4. The cell number decreased in a concentration-dependent manner following GEM treatment in KP4 cells, as observed in the case of MiaPaCa-2 cells (Figure 3c). However, no effect was observed beyond 0.1 μM GEM. Therefore, we used only 0, 0.01, and 0.1 μM concentrations of GEM for the qPCR experiments. The mRNA expression of CYP1B1 and CYP2A6 increased upon GEM treatment with increasing concentrations of GEM (Figure 3d).

**Figure 4:**
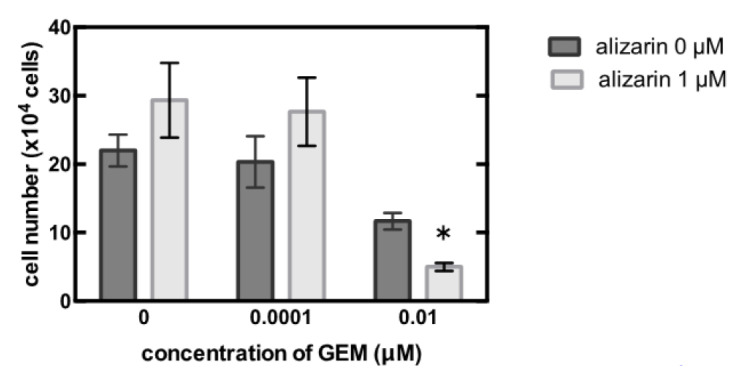
MiaPaCa-2 cells were treated with 0–0.01 µM gemcitabine (GEM) and 0 or 1 µM alizarin for 48 h, and the cell number was counted. Data represent mean ± SEM. *n* = 3. * *p* < 0.05.

Ultimately, we treated the MiaPaCa-2 cells with the CYP1B1 inhibitor, alizarin, which resulted in the significant inhibition of cell proliferation (Figure 4).

## 4. Discussion

Gemcitabine is a deoxycytidine analog, which, since its approval by FDA in 1996, has been used for the treatment of various tumors [17]. It is phosphorylated intracellularly by the deoxycytidine kinase to its active form, difluorodeoxycytidine triphosphate, which inhibits DNA synthesis by competing with deoxycytidine triphosphate (dCTP) for incorporation into nascent DNA strands, and then cell death occurs by means of apoptosis [18]. Gemcitabine also stimulates the activity of the intracellular deoxycytidine kinase, and it inhibits ribonucleotide reductase. It then reduces the intracellular pool of dNTPs as well as the enzyme deoxycytidine monophosphate deaminase, which is involved in gemcitabine degradation [19,20]. However, there are few reports on such metabolic activities that weaken the functional activity of gemcitabine. In this study, we conducted experiments using the PDX model to investigate the drug resistance of gemcitabine and analyzed the obtained samples by means of RNA-seq and gene set enrichment analysis (GSEA). The results of the analysis showed a possible relationship between monooxygenase activity and gemcitabine resistance.

CYP (cytochrome P450) is a large superfamily of highly conserved integral membrane proteins that are present in animals, plants, and various microorganisms [21,22]. The CYP system is expressed in all mammal tissues [23]. The CYP superfamily is primarily expressed in the liver, kidneys, and small intestine [24,25]. The CYP monooxygenase system is the cornerstone and a major catalyst in drug biotransformation reactions. It is involved in the metabolism of lipophilic endogenous and xenobiotic compounds and is responsible for transforming them into hydrophilic or polar compounds that can be easily excreted from the body [26]. The CYP1B1 gene is transcriptionally induced by polycyclic aromatic hydrocarbons via the aryl hydrocarbon receptor (AhR) complex [27,28]. The most potent of these AhR agonists for activating the transcription of CYP1B1 gene appears to be dioxin [28]. There are also some AhR-independent up-regulations of CYP1B1. CYP1B1 is also up-regulated by 17β-estradiol through estrogen receptor α [29]. CYP1B1 and CYP4F enzymes were up-regulated by the inflammatory cytokine interleukin-6 (IL-6), however most of the CYP1, CYP2, and CYP3 families were down-regulated [30,31,32]. The Wnt/β -catenin signaling pathway also invovles CYP1B1 up-regulation in endothelial cells [33].

CYP1B1 is expressed in normal tissue, but at very low levels compared to cancerous tissue. On the other hand, very high expression is observed at the protein level in tumor tissues [34,35]. Therefore, CYP1B1 is a tumor biomarker, and the inhibition of CYP1B1 activity is considered to be a therapeutic target for cancer chemoprevention and chemotherapy. Alizarin, purpurin, and 2,4,3′,5′-tetramethoxy-trans-stilbene (TMS) are known as the CYP1B1 inhibitor. Purpurin and TMS also inhibit the activities of CYP1A1 and CYP1A2 [36,37]. It was shown that alizarin strongly inhibited the activities of CYP1A1, CYP1A2, and CYP1B1 and weakly suppressed CYP2A6 and CYP2E1 [36]. However, CYP1A1, CYP1A2, and CYP2E1 were not detected in this study. Hence, alizarin acted as a CYP1B1 and CYP2A6 inhibitor here.

While there only a few reports have been published regarding the direct relationship between CYP1B1 and GEM, there are several studies on CYP1B1 and chemotherapy. For instance, Zhu et al. reported that the combination of CYP1B1 inhibitor and the anti-cancer drug paclitaxel prevented the proliferation of epithelial ovarian cancer cells in vitro and in vivo, indicating that CYP1B1 enhances the chemoresistance of ovarian cancer [38]. Other in vitro studies have suggested that CYP1B1 increases the resistance of V79 cells to docetaxel (DTX) and antagonizes the anticancer effects of DTX [39]. A similar result is shown MCF-7 cells with DOX [40]. Moreover, CYP1B1 silencing is significantly reduced cisplatin resistance in cisplatin-resistant non-small cell lung cancer cells [41]. These previous anti-cancer drug studies and our gemcitabine data show that the co-administration of CYP1B1 inhibitors and anti-cancer drugs decreases drug resistance and ameliorates the outcome of anti-cancer therapy [42]. However, the precise mechanisms underlying these observations remain unknown. Understanding the efficiency of each inhibitor in different cancers and resistance to each anti-cancer drug requires further research, including the analysis of another specific CYP inhibitor, CYP activity assays, and the evaluation of the CYP induction protein level.

## 5. Conclusions

In this study, we successfully established seven PDX lines of pancreatic cancer with different sensitivities to GEM. Furthermore, we found that the genes involved in monooxygenase activity were upregulated in a concentration-dependent manner in GEM-treated cells in vitro. Finally, we deduced that CYP1B1 inhibition had also accelerated the decrease in cell number upon GEM treatment. These results suggest that the CYP1B1 pathway is involved in GEM resistance.

## Figures and Tables

**Table 1:** Gene set enrichment analysis (GSEA). In all, 288 sets of genes were analyzed. Top 34 gene sets are shown. As seen, the highest gene set was associated with monooxygenase activity. ES: the number of genes in the gene set after filtering out those genes not in the expression dataset. NES: normalized enrichment score; that is, the enrichment score for the gene set after it has been normalized across analyzed gene sets. NOM *p*-value: nominal *p* value; that is, the statistical significance of the enrichment score. The nominal *p*-value is not adjusted for gene set size or multiple hypothesis testing; therefore, it is of limited use in comparing gene sets. FDR q-value: false discovery rate; that is, the estimated probability that the normalized enrichment score represents a false positive finding. FWER *p*-value: family wise-error rate; that is, a more conservatively estimated probability that the normalized enrichment score represents a false positive finding. As the goal of GSEA is to generate hypotheses, the GSEA team recommends focusing on the FDR statistic. RANK AT MAX: the position in the ranked list at which the maximum enrichment score occurred. The more interesting gene sets achieve the maximum enrichment score near the top or bottom of the ranked list; that is, the rank at max is either very small or very large. LEADING EDGE: displays the three statistics used to define the leading-edge subset. Tags: the percentage of gene hits before (for positive ES) or after (for negative ES) the peak in the running enrichment score. This indicates the percentage of genes that contributed to the enrichment score. List: the percentage of genes in the ranked gene list before (for positive ES) or after (for negative ES) the peak in the running enrichment score. This indicates that the enrichment score is attained in the list. Signal: the enrichment signal strength that combines the two previous statistics: (Tag %) (1 – Gene %) (N/(N-Nh), where N is the number of genes in the list and Nh is the number of genes in the gene set. If the gene set is entirely within the first Nh positions in the list, then the signal strength is maximal or 100%. If the gene set is spread throughout the list, the signal strength decreases to 0%. These statistics describe the leading-edge subset of a single gene set. Leading edge analysis was used to analyze the overlap between multiple leading-edge subsets.

Name	Size	ES	NES	NOM *p*-Value	FDR q-Value	FWER *p*-Value	Rank at Max	Leading Edge
ANION_TRANSMEMBRANE_TRANSPORTER_ACTIVITY	56	−0.4869358	−2.101442	0	0.033455465	0.051	2391	tags = 39%, list = 15%, signal = 46%
EXTRACELLULAR_MATRIX_STRUCTURAL_CONSTITUENT	26	−0.58796436	−2.0886946	0	0.024774604	0.057	2216	tags = 46%, list = 14%, signal = 53%
COLLAGEN	23	−0.603812	−2.0409071	0	0.028811615	0.085	1935	tags = 43%, list = 12%, signal = 49%
INORGANIC_ANION_TRANSMEMBRANE_TRANSPORTER_ACTIVITY	19	−0.54800725	−1.8485612	0.006849315	0.13804246	0.414	1759	tags = 37%, list = 11%, signal = 41%
PEPTIDE_RECEPTOR_ACTIVITY	44	−0.4471173	−1.7894889	0.003921569	0.19005722	0.582	1946	tags = 36%, list = 12%, signal = 41%
ANION_CATION_SYMPORTER_ACTIVITY	16	−0.56837684	−1.7396722	0.021341464	0.24313329	0.713	1378	tags = 38%, list = 8%, signal = 41%
SULFOTRANSFERASE_ACTIVITY	22	−0.49270993	−1.701363	0.009646302	0.27869517	0.805	1937	tags = 36%, list = 12%, signal = 41%
PROTEOGLYCAN_METABOLIC_PROCESS	17	−0.53180295	−1.6986526	0.01618123	0.25181675	0.811	1768	tags = 29%, list = 11%, signal = 33%
LIGAND_GATED_CHANNEL_ACTIVITY	35	−0.44609684	−1.6825303	0	0.25340274	0.833	1930	tags = 34%, list = 12%, signal = 39%
ION_HOMEOSTASIS	115	−0.34891573	−1.6815822	0	0.23189601	0.834	2002	tags = 28%, list = 12%, signal = 31%
HORMONE_METABOLIC_PROCESS	29	−0.4490808	−1.6701156	0.015444015	0.23098724	0.863	1011	tags = 24%, list = 6%, signal = 26%
CORTICAL_CYTOSKELETON	18	−0.5224792	−1.6676438	0.015625	0.21772708	0.869	2712	tags = 44%, list = 17%, signal = 53%
HEPARIN_BINDING	20	−0.5068244	−1.6632581	0.014492754	0.20953959	0.877	1576	tags = 40%, list = 10%, signal = 44%
AMINO_ACID_TRANSPORT	25	−0.47122175	−1.658062	0.013793103	0.20178199	0.885	1581	tags = 28%, list = 10%, signal = 31%
TRANSFERASE_ACTIVITY_TRANSFERRING_SULFUR_CONTAINING_GROUPS	26	−0.46915933	−1.6546559	0.010830325	0.19338286	0.891	1937	tags = 31%, list = 12%, signal = 35%
EXTRACELLULAR_MATRIX_PART	52	−0.39951557	−1.6543405	0.004149378	0.18208581	0.891	1935	tags = 31%, list = 12%, signal = 35%
ION_TRANSMEMBRANE_TRANSPORTER_ACTIVITY	263	−0.30467132	−1.6533009	0	0.1726972	0.892	2264	tags = 26%, list = 14%, signal = 30%
AMINE_TRANSPORT	34	−0.42308894	−1.6444278	0.01171875	0.17343472	0.902	1581	tags = 29%, list = 10%, signal = 32%
POLYSACCHARIDE_BINDING	29	−0.45253384	−1.633435	0.014981274	0.17863564	0.921	1576	tags = 34%, list = 10%, signal = 38%
CHEMICAL_HOMEOSTASIS	138	−0.33011207	−1.6284368	0	0.17518602	0.931	2002	tags = 28%, list = 12%, signal = 31%
SYNAPTOGENESIS	18	−0.5083024	−1.6254768	0.013745705	0.17086776	0.932	2646	tags = 33%, list = 16%, signal = 40%
EXTRACELLULAR_MATRIX	91	−0.34340963	−1.6218148	0	0.1676017	0.933	2251	tags = 32%, list = 14%, signal = 37%
PROTEINACEOUS_EXTRACELLULAR_MATRIX	90	−0.3495134	−1.6129957	0	0.17004865	0.946	2251	tags = 32%, list = 14%, signal = 37%
NEUROTRANSMITTER_BINDING	49	−0.3874927	−1.6098273	0.015444015	0.16672221	0.95	1913	tags = 31%, list = 12%, signal = 35%
STEROID_BIOSYNTHETIC_PROCESS	22	−0.4757495	−1.6048489	0.032467533	0.16537002	0.956	2297	tags = 45%, list = 14%, signal = 53%
G_PROTEIN_COUPLED_RECEPTOR_ACTIVITY	170	−0.3093993	−1.5869783	0	0.18080884	0.975	2000	tags = 25%, list = 12%, signal = 29%
PATTERN_BINDING	35	−0.40934092	−1.5851388	0.010830325	0.17625666	0.977	2241	tags = 37%, list = 14%, signal = 43%
REGULATION_OF_BODY_FLUID_LEVELS	55	−0.36799893	−1.5495309	0.00952381	0.22113751	0.993	2331	tags = 31%, list = 14%, signal = 36%
GLYCOSAMINOGLYCAN_BINDING	27	−0.42942247	−1.5400821	0.02734375	0.22773191	0.994	1576	tags = 33%, list = 10%, signal = 37%
NEUROTRANSMITTER_RECEPTOR_ACTIVITY	46	−0.38586268	−1.5308675	0.023809524	0.234069	0.996	1913	tags = 30%, list = 12%, signal = 34%
GLUTAMATE_RECEPTOR_ACTIVITY	18	−0.47083804	−1.527544	0.038709678	0.2313607	0.997	705	tags = 22%, list = 4%, signal = 23%
CATION_HOMEOSTASIS	98	−0.31595644	−1.5160195	0.011428571	0.24062736	0.997	2002	tags = 27%, list = 12%, signal = 30%
ACETYLCHOLINE_BINDING	17	−0.47908843	−1.5119975	0.04452055	0.23989336	0.997	2446	tags = 41%, list = 15%, signal = 48%

## Data Availability

The datasets generated during and/or analysed during the current study are available from the corresponding author on reasonable request.
